# An explainable and federated deep learning framework for skin cancer diagnosis

**DOI:** 10.1371/journal.pone.0324393

**Published:** 2025-07-16

**Authors:** Shuvo Biswas, Sajeeb Saha, Muhammad Shahin Uddin, Rafid Mostafiz

**Affiliations:** 1 Department of Information and Communication Technology, Mawlana Bhashani Science and Technology University, Tangail, Bangladesh; 2 Department of Computer Science and Engineering, The People’s University of Bangladesh, Dhaka, Bangladesh; 3 Institute of Information Technology, Noakhali Science and Technology University, Noakhali, Bangladesh; Kafkas University: Kafkas Universitesi, TÜRKIYE

## Abstract

Skin cancer (SC) is the most prominent form of cancer in humans, with over 1 million new cases reported worldwide each year. Early identification of SC plays a crucial role in effective treatment. However, protecting patient data privacy is a major concern in medical research. Therefore, this study presents a smart framework for classifying SC leveraging deep learning (DL), federated learning (FL) and explainable AI (XAI). We tested the presented framework on two well-known datasets, ISBI2016 and ISBI2017. The data was first preprocessed by several techniques: resizing, normalization, balancing, and augmentation. Six advanced DL algorithms—VGG16, Xception, DenseNet169, InceptionV3, MobileViT, and InceptionResNetV2—were applied for classification tasks. Among these, the DenseNet169 algorithm obtained the highest accuracy of 83.3% in ISBI2016 and 92.67% in ISBI2017. All models were then tested in an FL platform to maintain data privacy. In the FL platform, the VGG16 algorithm showed the best results, with 92.08% accuracy on ISBI2016 and 94% on ISBI2017. To ensure model interpretability, an XAI-based algorithm named Local Interpretable Model-Agnostic Explanations (LIME) was used to explain the predictions of the proposed framework. We believe the proposed framework offers a dependable tool for SC diagnosis while protecting sensitive medical data.

## 1. Introduction

In recent years, skin cancer (SC) cases are rapidly increasing in some countries. If identified at an early stage, this type of deadly disease is curable [1]. Consequently, swift detection is a promising method for lowering the death rate from SC [2,3]. To aim to forecast SC treatments with a mistake that is less than that feasible for people [4], as there are diverse possible health risks involved with them, apart from morbidity and cost. Minor errors occur in these technologies, and detection results are sometimes inaccurate. As a result, a strong computer-aided diagnostic system can help doctors prevent errors. Preprocessing, feature extraction, feature interpretability, data privacy, and prediction are common methods for early SC prediction [5]. Abnormalities and tumors in the blood and lymphatic system can result from aberrant lesion cell division. Both melanoma and benign lumps can be produced through the lymphatic system of the body, while benign lumps are confined and fail to grow widely [6]. As SC is visible with the bare eye, this type of disease is quicker to identify than melanoma.

Artificial intelligence (AI)-based algorithms, specifically deep learning (DL) algorithms, have exhibited results in accurately categorizing and identifying SC in the health sector [7]. Recently, DL methods have been used in various fields like remote sensing technologies [8–10]. Researchers used several advanced and lightweight DL algorithms like vision transformers to detect and classify SC automatically [11]. Despite their potential, these algorithms typically require vast amounts of data for effective training, which is often rare in most healthcare environments due to limited access to extensive patient records. One of the main pitfalls is proposing a strong and accurate DL-based framework that can properly differentiate different types of SC from other skin-related diseases [12]. To build a generalizable DL-based framework, it is important to use a variety of datasets to evaluate these models.

Federated Learning (FL) is an advanced machine learning (ML) technique that allows DL-based algorithms to be trained on data from diverse servers without transmitting the data to a central platform [13]. Instead, the DL-based algorithm is trained locally on each server, which helps protect sensitive information. This approach is applicable in healthcare environments, i.e., melanoma SC identification, where privacy preservation is a major concern. By using FL, hospitals can collaborate to build a more accurate and generalized framework without exposing patients’ information. However, implementing FL in practical life can face diverse challenges: differences in data quality between hospitals, the need for stable network connections to manage communication, and limitations in hardware that may affect processing power. Additionally, ensuring compliance with healthcare regulations and combining the system into existing workflows are important aspects that must be solved for successful deployment. FL offers several advantages, particularly in privacy-sensitive domains like healthcare [14]. By enabling collaborative model training without sharing raw patient data, FL enhances data privacy and security [15]. Additionally, FL allows models to leverage diverse datasets from multiple hospitals, leading to more generalized and robust results. However, FL encounters several challenges, including high communication overhead, as frequent model updates between clients and the central server require significant bandwidth [16]. Data heterogeneity across clients can lead to inconsistent learning, making it difficult to train a global model. Moreover, FL requires computationally capable edge devices, which may not always be available in resource-constrained settings. Finally, integrating FL into real-world clinical workflows demands careful coordination between hospitals, IT infrastructure upgrades, and compliance with evolving regulations. The main contributions of this manuscript are

- Evaluate the generalizability of the proposed framework using two datasets named ISBI16 and ISBI2017.
- Apply six advanced DL-based algorithms named InceptionResNetV2, VGG16, MobileViT, Xception, Densenet169, and InceptionV3 to automatically classify skin cancer.
- Evaluated these algorithms in the federated learning environment to ensure data privacy during skin cancer classification.
- Finally, an XAI-based algorithm named Local Interpretable Model-Agnostic Explanations (LIME) is employed to provide local interpretation of the proposed framework.

The structure of the article is as follows. Related works is covered in **Section 2**. The materials and methods of the paper are provided in **Section 3**. **Section 4** shows the experiments, results analysis, and discussion. Finally, the conclusion is found in **Section 5.**

## 2. Related works

Researchers have enlightened various methods and methodologies for SC classification in their published articles. Some authors presented novel methods, but some have major limitations in their methodology. Table 1 shows a summary of previously published articles that contains the dataset, strengths, and limitations of each work.

**Table 1:** Comparison table of the published paper.

Author/Year	Dataset	Strengths	Limitations
Yu. *et al* [17]/2016	ISBI2016	A flexible framework used to effectively segment the skin lessions.	A slight drop in prediction accuracy occurs without segmentation, indicating some reliance on it.
Ali *et al* [18]/2017	ISBI2016	A LightNet framework that provides higher degree of specificity value.	Lack of model interpretability; Low sensitivity value.
Yu *et al* [19]/2018	ISBI2016	Proposed a unique approach by leveraging DL algorithms and FV algorithms.	Lack of data privacy and model transparency.
Romero *et al* [20]/2017	ISBI2016	A balanced framework that provides higher degree of precision and recall.	The performance can be increased by leveraging vision transformer based network.
Demir *et al* [21]/2019	ISIC archive	Provided outstanding performance using InceptionV3 classifier.	The classification rate is very low.
Jain *et al* [22]/2021	HAM10000	Obtained best accuracy (0.91) using Xception classifier.	Low classification rate; Privacy preserving is needed.
Khan *et al* [23]/2024	ISBI2016-ISIC2019, HAM10000	A combined network that provides best accuracy (0.93).	A series of fine-tuning layers are needed to improve the performance.
Gouda *et al* [24]/2022	ISIC2018	A novel data augmentation approach was developed that boosted the training time and classification rate.	Lack of feature interpretability and transparency.
Aljohani *et al* [25]/2022	ISIC2019	Provided best accuracy (0.761) using GoogleNet.	The performance of the proposed system may be improved using vision transformer based models.
Fraiwan *et al* [26]/2022	HAM10000	Obtained best accuracy (0.829) using DenseNet201.	Provides unsatisfactory F1 value (74.4%).
Gajera *et al* [27]/2023	ISBI2016	A combined framework that obtained outstanding performance (accuracy of 0.9833).	Lack of model generalizability.
Bassel *et al* [28]/2022	ISIC2019	Provided best accuracy of 90.9% using exception classifier.	Complex architecture that enhances the computational cost.
Keerthana *et al* [29]/2023	ISBI2016	A combined network (DenseNet201 + MobileNet + SVM) that obtained best accuracy of 88.02%.	Privacy-preserving is needed that makes the model more reliable and dependable.

In brief, this manuscript presents a reliable and dependable system for addressing the above challenges that are tabulated in Table 1. The proposed system addressed these challenges by training DL algorithms under an FL environment. The FL-based system provides data privacy by sharing models with other parties without data sharing. Finally, an XAI-based method is used in this work to make the proposed framework more transparent and understandable.

## 3. Materials and methods

**Fig 1:**
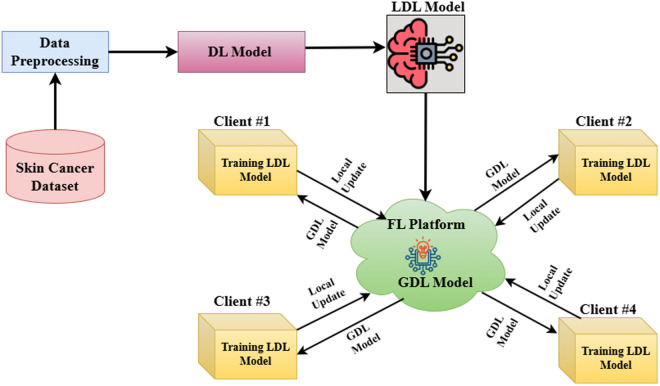
Conceptual diagram of the proposed system leveraging deep learning and federated learning. Here LDL means local deep learning model and GDL means global deep learning model.

This section explains the development procedures of the proposed framework for SC detection. The conceptual diagram of the proposed framework is shown in Fig 1. This process includes the following phases: data preprocessing, training a local DL (LDL) algorithm, training an LDL algorithm in an FL server as a global DL (GDL) algorithm, and implementing an XAI-based LIME method. Firstly, the dataset is resized and normalized into the proper format. Then a data balancing strategy named Synthetic Minority Over-sampling Technique (SMOTE) is used for balancing the imbalanced SC dataset and expanding the balanced dataset through data augmentation. After that, five LDL algorithms are used to differentiate benign cases from melanoma cases. To protect data privacy and further evaluation, these LDL algorithms are trained under the FL platform to make the GDL algorithm. These combined methodologies aim to enhance the classification rate as well as ensure data privacy. The working procedure of this work is summarized in Algorithm 1.

**Algorithm 1:** Algorithm for the proposed work.

***input:*** *Dataset: ISBI, [train_set S1:70%; valid_set S2:10%; test_set S3:20%]* * bs ← batch_size* * ep ← epoch* * op ← optimizer* * lr ← learning_rate* * mbs ← mini_batch_size* ***output:*** *Weight of DL algorithm* ***start:*** * 1: Resize each data into 224* × *224 and normalized into range [0, 1];* * 2: Apply SMOTE method for data balancing and used augmentation technique to expand training data;* * 3: sed six DL models: DLM*_*DenseNet169*_*, DLM*_*InceptionV3*_*, DLM*_*MobileViT*_*, DLM*_*Xception*_*, DLM*_*VGG16*_*, and DLM*_*InceptionResNetV2*_ *for feature extraction;* * 4: Then used a CNN layer: CNN*_*global_average_pooling*_ *for flattening the extracted features;* * 5: Three CNN layers: CNN*_*dense*_*, CNN*_*dropout*_*, and CNN*_*softmax*_ *are used for fine-tuning purposes;* * 6: Set the training aurguments: bs, ep, op, lr, and mbs;* * 7: Train the DL algorithm for calculating the overall weights;* * 8:* ***for*** *k = 1 to r* ***do*** * 9: Select mini_batch_size ← mbs;* * 10: Forwardpropagation with binary_loss_function ← blf;* * 11: Backpropagation with model weight ← wt;* * 12:* ***end for*** ***stop***

### 3.1 Experimental data

**Fig 2:**
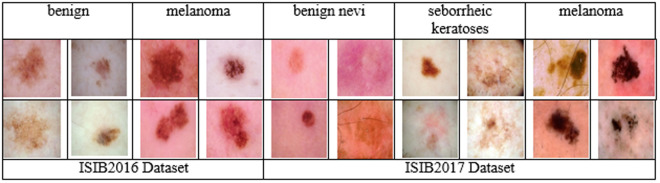
Example images from ISIB2016 and ISIB2017 datasets.

In this work, two benchmark datasets named ISBI2016 [30] and ISBI2017 [31] are used to differentiate benign cases from melanoma cases. The International Symposium on Biomedical Imaging (ISBI) presented two publicly challenging datasets named ISBI2016 and ISBI2017 for skin lesion analysis toward melanoma identification that were hosted on the International Skin Imaging Collaboration (ISIC) archive. These two datasets are used to ensure the proposed model’s generalizability and fully reflect the diversity of SC characteristics across different regions and populations. The first dataset (ISBI2016) was composed of (n = 1279) skin cases, where (n = 900) samples were for training and the remaining (n = 379) samples were for testing. On the other hand, the second dataset (ISBI2017) was partitioned into 3 directories: detection (4 classes), segmentation, and classification (3 classes). In this experiment, we used the classification directory to predict whether a lesion is melanoma or benign. This directory consists of (n = 2750) skin cases, where (n = 2000) are for training, (n = 150) are for validation, and (n = 600) are for testing. This dataset contains three types of cancer cases: benign nevi (1372 train, 393 test, and 78 validation), seborrheic keratoses (254 train, 90 test, and 42 validation), and melanoma (374 train, 117 test, and 30 validation). These two datasets contain various sizes of images ranging from 1022 × 767 to 4288 × 2848 pixels. Each melanoma sample was denoted as 1 and benign as 0. Fig 2 shows example images from the ISBI2016 and ISBI2017 datasets.

### 3.2 Data preprocessing

Before training the DL algorithms, many preprocessing steps are performed. Firstly, we resized the collected images into 224 × 224 pixels with RGB channels. Then the pixel score of each sample is normalized into the range 0 to 1. Challenges like a small dataset and uneven class distribution can affect the model’s accuracy. However, the collected dermoscopic images were imbalanced. To tackle this important issue, the SMOTE operation is used to produce synthetic images for the minority label. This operation produces new samples randomly by adding samples from the majority label [32]. After the data balancing operation, the training set in the ISIB2016 dataset is enhanced from 810 to 1308 samples.

**Fig 3:**
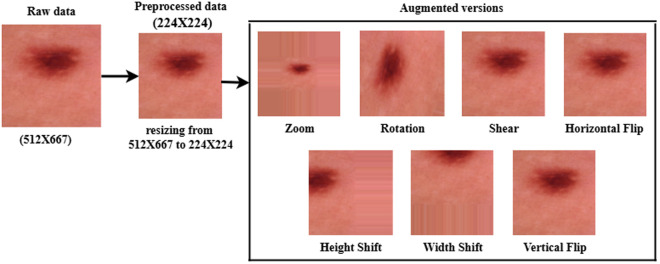
Example for a specific image that illustrates the raw, preprocessed, and augmented versions.

**Fig 4:**
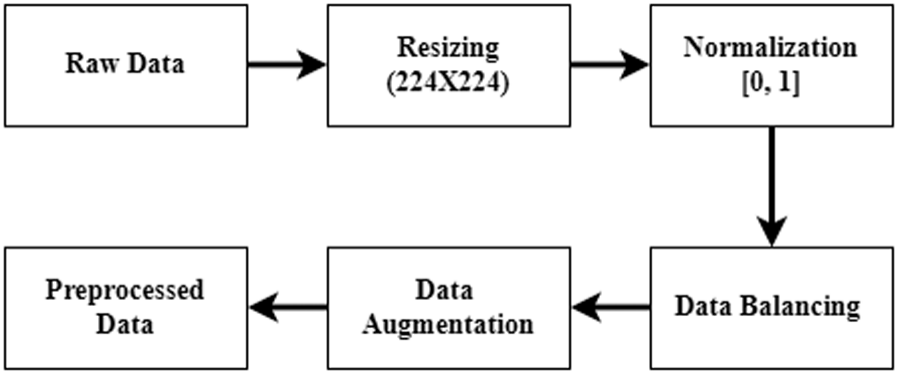
Flow diagram for data preprocessing stages.

In the DL method, a large number of training data is needed to train a DL algorithm. However, in the medical sector, the number of medical images is insufficient. To tackle this issue, we used a data augmentation operation. In this study, we used seven data augmentation operations with value: rotating with a value of 90 degrees, height shifting with a value of 0.2, width shifting with a value of 0.2, zooming with a value of 2, horizontal flipping with a value true, vertical flipping with the value true, and shearing with value 0.4. After performing a data augmentation operation, the training set in the ISIB2016 dataset is enhanced from 1308 to 10464 dermoscopy skin images, where 5232 are benign and 5232 are melanoma cases. Conversely, the validation set is enhanced from 146 to 1168, with 584 benign cases and 584 melanoma cases. Table 2 shows data distribution after performing data splitting, balancing, and augmenting operations. Fig 3 shows an example of a specific image that illustrates the raw, preprocessed, and augmented versions. Fig 4 represents the flow diagram outlining the data preprocessing steps in the SC classification task.

**Table 2:** Number of samples of ISBI2016 dataset after splitting, balancing and augmenting operations.

			After splitting		After balancing		After augmentation	
Label	Train	Test	Train (90%)	Valid (10%)	Train	Valid	Train	Valid
Melanoma	173	75	156	17	654	73	5232	584
Benign	727	304	654	73	654	73	5232	584
Total	900	379	810	90	1308	146	10464	1168

### 3.3 Fine-tuned DL network

**Fig 5:**
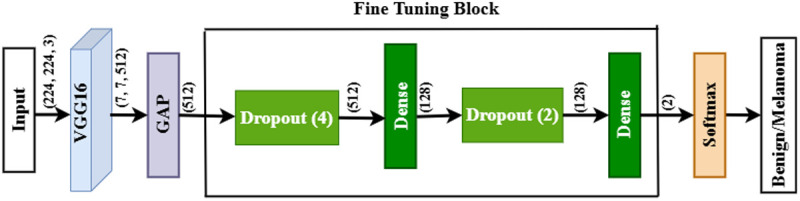
The schematic diagram of a fine-tuned DL network. Here, GAP indicates the global_average_pooling layer. The fine-tuning block consists of four CNN layers, two dropout layers, and two dense layers. Softmax activation functions classify the cancer types.

This section describes the construction process of a fine-tuned DL network. A fine-tuned DL model consists of three components: a DL-based CNN algorithm, a global-average-pooling (GAP) layer, and a fine-tuning (FT) block. Each CNN algorithm retrieved DL features from the dataset and fed these retrieved features into the GAP layer. The GAP layer converted these retrieved features into a feature map (FM) and flattened this FM into a one-dimensional (1D) FM. After that, an FT block is used to process this 1D FM for final classification tasks. An FT block consists of four DL layers: two dropout layers and two fully connected (FC) layers. The final FC layer has a softmax activation function (SAF) that classifies the cancer type. The conceptual diagram of a fine-tuned DL model is depicted in Fig 5. The next subsections briefly explain each component step by step.

#### (i) InceptionV3.

The InceptionV3 [33] CNN model is a modified version of the GoogleNet model. This algorithm has an Inception section that integrates convolution modules with diverse sizes. These modules help this algorithm retrieve features at several phases. However, the stacked Inception modules permit this algorithm to retrieve more deep features. This algorithm improves the prediction results during training time by combining several CNN layers: fully connected, convolutional, pooling, and extra layers.

#### (ii) Xception.

Xception [34] is a modified version of the Inception CNN model. This network substitutes the traditional convolutional layer (TCL) with a depth-wise separable layer (DSL) for separately handling cross-channel and spatial data. However, this network has 36 convolutional and 11 depth-wise convolutional CNN layers. In the XceptionNet model, TCL calculates probabilities from each input channel, and DSL extracts features based on the calculated probability scores. Finally, this model predicts the target objects based on these extracted features.

#### (iii) VGG16.

VGG16 [35] The DL algorithm was introduced by the Visual Geometry Group at Oxford University. It has 16 CNN layers, 13 convolutional layers, and three fully connected (FC) layers. Convolutional layers are utilized for DL feature extraction from the input data, and FC layers are utilized for final classification. This algorithm mainly performs several image analysis operations: classification, detection, and segmentation.

#### (iv) InceptionResNetV2.

Inception ResNetV2 [36] is a modified version of the Inception and ResNetV2 models. This algorithm was simulated on a large database named ImageNet that contains more than 1 M (million) images with 1000 different classes. This novel DL algorithm has 164 CNN layers and takes 299 × 299 pixel images as its input data. Due to learning different features from different classes, this algorithm is able to classify different images accurately.

#### (v) DenseNet169.

The DenseNet169 [37] CNN algorithm consists of 169 layers that have been pre-trained on 1 M (million) images from the ImageNet dataset. This algorithm can identify 1000 different target classes, as it already learned a large number of high-resolution features from several types of images. The DenseNet169 algorithm takes 224 × 224 pixel images as input to perform the final classification task.

### 3.4 Final classification process

This section presents the final classification process or simply fine-tuning (FT) process. This process is performed through three CNN layers: global_average_pooling2D (GAP), dropout, and dense/fully connected (FC). The GAP layer combines the extracted features from the above CNN algorithms to create a feature map (FM). This FM then passes into other CNN layers.

In the FT process, two dropout [38] layers are used to reduce the overfitting issue, where the first layer discards 20% of the data and the second discards 40% of the data. On the other hand, the first FC [39] layer connects all neurons to each other. The first FC layer used the ReLU activation function to reduce the output shape. Finally, the second FC layer with the SAF categorizes skin cancer types. That’s why the output shape of this CNN layer is two (benign/melanoma). The mathematical formula for the SAF is given by equation 1. Table 3 lists the functional layers of the VGG16 algorithm with output and parameters.

**Table 3:** Summary of different functional layers of VGG16 model with parameters.

Layer (type)	Output Shape	Param #
input_layer_1 (InputLayer)	(None, 224, 224, 3)	0
vgg16 (Functional)	(None, 7, 7, 512)	14,714,688
global_average_pooling2d (GlobalAveragePooling2D)	(None, 512)	0
dropout (Dropout)	(None, 512)	0
dense (Dense)	(None, 128)	65,664
dropout_1 (Dropout)	(None, 128)	0
dense_1 (Dense)	(None, 2)	258

**Total params:** 14,780,610 (56.38 MB).

**Trainable params:** 7,145,346 (27.26 MB).

**Non-trainable params:** 7,635,264 (29.13 MB).


$$\text{SAF}(\text{y}{)}_{\text{r}}=\frac{\exp\left(y_{r}\right)}{\sum_{n=1}^{m}\exp\left(y_{r}\right)}$$ (1)

### 3.5 Federated learning approach

Flower FL [40] paradigm is used in this experiment to create a privacy preserving environment. Six popular DL algorithms are selected as the local DL (LDL) algorithm for the pneumonia prediction task under the FL environment. In the FL mechanism, at first, the central platform converts each LDL model into the global DL (GDL) algorithm and waits for the final update. After that, agents connect to the central platform and download GDL algorithms for training their private data. Then the agents provide updates of the GLD algorithms to the central platforms without sharing their private data. Finally, the central platform integrates all updates using the FedAvg [41] method. The whole FL mechanism is calculated using Equation 2.


$$G_{r+1}^{u}=\frac{1}{a_{n}}\sum_{n=1}^{a_{n}}{w_{n}*G}_{r}^{v}$$ (2)

Here, $G_{R+1}^{v}$ is the global update of the GDL algorithm at moment *r + 1*, $a_{n}$ indicates the total number of agents during the FL mechanism, $w_{n}$ is the weights for each agent, and $G_{r}^{v}$ means to the global parameters at moment *r*.

However, the selection of optimal hyperparameters enhances the model performance and ensures efficient training. We select the sparse_categorical_crossentropy loss function during model training, as it is well-suited for binary-class classification problems where the FL learning approach is applied. The number of epochs was set to 10 after experimenting with the model with different values, as after 10 epochs the model makes overfitting. A learning rate (LR) of 0.0001 was selected from the search space, as it provided a stable convergence while avoiding vanishing or exploding gradients. The Adam optimizer was used due to its adaptive LR mechanism. Additionally, a batch size (BS) of 32 was found to be optimal, as it offered a trade-off between computational cost and model performance. These hyperparameters were fine-tuned based on experimental validation to enhance the model’s accuracy and robustness. The hyperparameter settings of each model with optimal value are given in Table 4.

**Table 4:** Hyperparameter setting for the FedAvg approach with optimal value.

Hyperparameter	Search Space	Optimal value
Loss function	[binary_crossentropy, **sparse_categorical_crossentropy**]	sparse_categorical_crossentropy
BS	[16,32, 64]	32
Optimizer	[**Adam**]	Adam
LR	[0.01, 0.001, **0.0001**, 0.00001]	0.0001
Epoch	[5,10,20,30]	10

### 3.6 Explainable artificial intelligence (XAI) approach

Explainable AI approach has shown great improvement over the traditional DL methods [42], where transparency and trustworthiness of the DL algorithm are needed [43–45]. The XAI method enhances the transparency, trustworthiness, and interpretability of the DL algorithms. As the traditional DL algorithms are black-box in nature, it is difficult to understand how they arrive at final consequences. Recently, XAI-based smart approaches like LIME have been used to address this pitfall that transforms the black-box model into a white-box model. This method highlights the important regions of an image that influence the final predictions. The importance of XAI lies in building trust in the proposed system and ensuring transparency and interpretability of the model’s decision, especially in critical applications such as medical diagnostics and security surveillance. In the realm of image classification, integrating XAI as an original contribution enhances model interpretability, facilitates performance improvements by identifying misclassifications, and helps medical specialists to make early diagnoses. Additionally, in FL, XAI plays a key role in understanding how local datasets impact GDL algorithm training, ensuring fairness and accountability across decentralized clients. By incorporating XAI, AI systems become more transparent, robust, and aligned with human reasoning, making them more trustworthy and practical for real-world deployment.

#### (i) Local interpretable model-agnostic explanations.

In the context of XAI, LIME [46] XAI technique allows a transparent explanation of an image being examined (*z ∈* ^*s*^), a binary activation set (*z∈ {0, 1}*^*s*^) was needed to evaluate the area of super-pixels. In LIME method, *f ∈  F*, with subset of feature *{0, 1}*^*s*^, was apply to show the explainable features *f*. However, it is not possible to understand the explanation of each feature in *f ∈ F*, that’s why *(f)* was implement to see the complexity of the feature interpretation. The feature interpretation of the LIME method is calculated by equation 3.


$$Accuracy(A)=\frac{TP+TN}{TP+TN+FP+FN}$$ (3)

Here, ξ*(z):* ℝ^*k*^ *→* ℝ means the predicted outcomes that *z* belongs to *{1, 2}* cases, and *𝜋*_*z*_*(y)* shows how closely connected two samples *y* and *z*. The fidelity map, *ω(u, v,* 𝜋_*z*_*)*, finds how much *u* fluctuates from *v* in area *𝜋*_*z*_. Here, the value of *δ(f*) is used to enhance the feature explanation by reducing the fidelity map.

## 4. Results and discussion

### 4.1 Hardware and software setup

This section outlines the experimental setup used to evaluate the DL algorithms. The DL algorithms were implemented using the Keras DL library integrated with the Python library. Experiments were conducted on the Google Colab workstation, Tesla-R90 GPU, an Intel Core i7-27300R CPU running at 3.8 GHz, and 32 GB of RAM. The Colab workstation provided 164 MB of cache, 512 GB of disk size, and a maximum session utilization of 12 Hr. Table 5 provides the versions of the core libraries used in this work.

**Table 5:** Versions of the core libraries used in this work.

Library	Version
Python	3.11.11
TensorFlow	2.18.0
Keras	3.8.0
NumPy	2.0.2
Pandas	2.2.2
Scikit-learn	1.6.1
Matplotlib	3.10.0
OpenCV	4.11.0
Seaborn	0.13.2

### 4.2 Performance indicators

This section presents the mathematical formulas for several performance indicators, such as accuracy, Matthews’s correlation coefficient (MCC), ROC-AUC, precision, Cohen’s kappa (CK), specificity, and F1-score. The corresponding equations for these metrics are provided as Equations (4) to (9).


$$Accuracy(A)=\frac{TP+TN}{TP+TN+FP+FN}$$ (4)

$$\text{MCC}=\frac{\left(TP\times TN)-(FP\times FN\right)}{\sqrt{\left(TP+FP)(TP+FN)(TN+FP)(TN+FN\right)}}$$ (5)

$$Specificity(S)=\frac{TN}{TN+FP}$$ (6)

$$Precision(P)=\frac{TP}{TP+FP}$$ (7)

$$\text{CK}=\frac{observedvalue-expectedvalue}{1-expectedvalue}$$ (8)

$$F1-score(F)=2\times \frac{PRE\times REC}{PRE+REC}$$ (9)

The Receiver Operating Characteristic (ROC) curve is a graphical representation that illustrates the trade-off between the True Positive Rate (TPR) and the False Positive Rate (FPR). Here, FN means false negative, TN means true negative, TP means true positive, and FP means false positive.

### 4.3 Results

The proposed system combines the DL algorithms for predicting SC with FL to ensure data privacy. This combined system utilizes the benefits of both methods, providing a dependable and effective tool in the medical sector. This system obtained high accuracy and specificity rates, exhibiting its efficiency in predicting several skin-related diseases. In this section, we compared centralized and FL approaches using multiple DL algorithms on ISBI datasets. Firstly, we evaluated each DL algorithm on the ISIB2016 and ISIB2017 datasets without FL, which is called the centralized approach. Similarly, these algorithms are evaluated on the FL environment using both datasets, which is called the decentralized or FL approach. The attained outcomes are described in this part, in which we investigate three methods: (i) the centralized method, where the full skin image data is trained utilizing the five DL algorithms picked to conduct this experiment (Section 3.3); (ii) a FedAvg-based FL method combination of five DL algorithms, where the entire skin image data is trained utilizing the GDL model to optimize the classification performance; and (iii) a FedProx-based FL method is used to compare with the results of the FedAvg-based approach.

#### (i) Experiment 1: Centralized method (without FL).

In the centralized approach, we directly utilized the five DL algorithms picked in section 3.3. The simulated results of these algorithms using the ISIB2016 dataset are tabulated in Table 6. In Table 6, the DenseNet169 algorithm attained the highest accuracy of 0.833, and the VGG16 algorithm obtained the lowest accuracy of 0.78 but attained the highest precision of 0.977 and specificity of 0.98.

**Table 6:** Simulated results of different DL algorithms using ISBI2016 dataset in centralized method.

Algorithm	A (%)	MCC (%)	S (%)	CK (%)	P (%)	AUC (%)	F (%)
InceptionResNetV2	81.3	64.1	92.0	62.7	89.8	81.3	79.1
DenseNet169	83.3	69.4	97.3	66.7	96.3	83.3	80.6
Xception	80.7	63.9	94.7	61.3	92.6	80.7	77.5
InceptionV3	80.0	64	97.3	60	95.9	80	75.8
VGG16	78.0	61.5	98.7	56	97.7	78	72.3

Similarly, the simulated results of these algorithms using the ISIB2017 dataset are tabulated in Table 7. In Table 7, the DenseNet169 algorithm attained the highest accuracy of 92.67%, and the MobileViT algorithm obtained the lowest accuracy of 87.67% but attained the highest specificity of 98.47%.

**Table 7:** Simulated results of different DL algorithms using ISBI2017 dataset in a centralized approach.

Algorithm	A (%)	MCC (%)	S (%)	CK (%)	P (%)	AUC (%)	F (%)
InceptionResNetV2	87.83	73.15	98.22	71.13	95.27	83.17	79.44
DenseNet169	92.67	84.21	92.37	84.07	86.55	92.80	89.77
Xception	92.17	82.70	98.47	81.95	96.51	89.33	87.60
MobileViT	87.67	72.87	98.47	70.63	95.86	82.81	78.98
InceptionV3	92.17	83.49	90.84	83.15	84.48	92.76	89.29
VGG16	89.17	75.85	97.20	74.79	93.29	85.56	82.48

#### (ii) Experiment 2: FedAvg-based FL method.

In this experiment, we apply the FedAvg-based FL method on the full skin image data to get the best results over experiment 1. In this method, the central server shared the DL algorithm with four clients as a global DL model. After that, each client trained the DL algorithm as a local DL model. The classification results of each DL algorithm are listed in Table 8 and Table 9.

**Table 8:** Simulated results of different DL algorithms using ISBI2016 dataset in FedAvg approach.

Algorithm	A (%)	MCC (%)	S (%)	CK (%)	P (%)	AUC (%)	F (%)
InceptionResNetV2	89.18	57.31	98.35	53.68	88.64	71.91	65.55
DenseNet169	89.71	64.89	97.37	63.35	84.62	78.02	69.29
Xception	88.65	58.60	99.34	56.22	94.44	74.02	61.26
InceptionV3	88.13	59.11	96.38	57.72	78.85	75.52	64.57
VGG16	92.08	73.55	98.36	72.28	90.91	82.51	76.92

**Table 9:** Simulated results of different DL algorithms using ISBI2017 dataset in FedAvg approach.

Algorithm	A (%)	MCC (%)	S (%)	CK (%)	P (%)	AUC (%)	F (%)
InceptionResNetV2	81.83	59.85	98.47	54.79	94.55	74.36	65.62
DenseNet169	93.67	85.90	97.71	85.66	95.19	91.85	90.36
MobileViT	71.17	63.31	58.02	58.22	54.67	75.95	69.70
Xception	88.65	58.60	99.34	56.22	94.44	74.02	61.26
InceptionV3	92.67	83.72	97.96	83.23	95.53	90.29	88.60
VGG16	94.00	86.71	98.47	86.35	96.72	91.99	90.77

Table 8 shows the simulated results of each DL algorithm using the ISIB2016 dataset in the FedAvg-based FL approach. Here VGG16 algorithm attained the highest accuracy of 0.9208, and the InceptionV3 algorithm obtained the lowest accuracy of 0.8813.

Table 9 exhibits the simulated outcomes of all DL algorithms using the ISIB2017 dataset in the FedAvg-based FL approach. In Table 9, the VGG16 algorithm attained the highest accuracy of 94%, and the MobileViT algorithm obtained the lowest accuracy of 71.17%.

**Fig 6:**
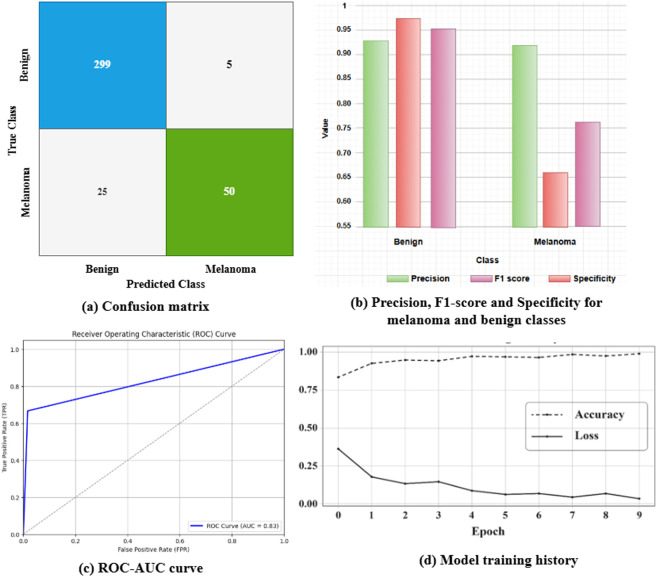
Evaluation metrics of FedAvg FL approach.

Fig 6 provides a detailed distribution of evaluation metrics of the proposed model in the FedAvg FL approach. Fig 6(a) shows the graph of precision, F1-measure, and specificity scores for benign and melanoma classes. In Fig 4(a), the melanoma class obtained the highest precision rate (91%) and the lowest specificity rate (67%); on the other hand, the benign class provided the highest specificity rate (98%) and the lowest precision rate (92%). This implies that the proposed system predicts melanoma cases more perfectly than benign cases. Fig 6(b) shows the confusion matrix (CM) of the proposed work. From CM, 299 melanoma images were predicted perfectly, and 5 samples were predicted inaccurately. Fig 6(c) shows the training history of the proposed model, where the accuracy increases over time. Similarly, the loss value decreases gradually, which implies that the model learns data effectively. Fig 6(d) shows the ROC-AUC curve for the proposed system that obtained the highest AUC score of 83% among all models. The number of parameters, prediction time, and GPU usage for the five core classification models are tabulated in Table 10.

**Table 10:** Summary of the no. of parameters, prediction time, and GPU usage of each classification model.

Model	No. of Parameters (millions)	Prediction Time (hours)	GPU Usage (MB)
DenseNet169	13.11	2.21	45.3423
VGG16	14.78	2.10	48.2930
InceptionResNetV2	54.77	4.53	92.7653
Xception	21.43	2.90	60.8753
InceptionV3	22.37	3.10	64.6713

#### (iii) Experiment 3: FedProx-based FL method.

In experiment 3, we investigated the simulated outcomes of employing the FedProx-based FL method attained at each algorithm in experiment 2 over the FedAvg-based FL approach. The goal is to find out the accuracy of the algorithms attained by employing the FedProx-based FL method. The simulated outcomes are tabulated in Table 11, and it is clear that the attained outcomes are higher than the centralized method in Experiment 1 (Table 7). Focusing on Table 11, in the final round, R10, the VGG16 algorithm obtained an accuracy of 93.89%, which is better than the centralized VGG16 algorithm but lower than the results of experiment 2 (Table 9). In round R10, the VGG16 model achieved the best performance in this experiment.

**Table 11:** Results of VGG16 algorithm using FedProx-based FL approach on ISIB2017 dataset. (Top results are highlighted in bold.).

Metrics	R1	R2	R3	R4	R5	R6	R7	R8	R9	R10
**A (%)**	86.39	87.22	89.17	89.44	91.67	91.94	93.06	93.33	93.61	**93.89**
**P (%)**	60.38	73.33	74.42	80.56	80.00	78.18	78.69	83.33	83.64	**85.19**
**F (%)**	56.64	48.89	62.14	60.42	72.73	74.78	79.34	78.95	80.00	**80.70**
**AUC (%)**	90.67	94.04	95.29	96.05	96.58	97.05	97.34	97.71	97.97	**98.19**

In summary, centralized training requires aggregating all data into a single location, which can result in the best performance due to entire data availability. However, this technique introduces significant concerns regarding data privacy, where sensitive patient data must be protected. In contrast, FedAvg allows collaborative framework training across distributed clients without sharing data. While it ensures privacy preservation and minimizes data leakage challenges, it can struggle with performance in the presence of non-identically distributed (non-IID) data across clients. To mitigate this, the FedProx-based FL method is presented in this study to limit client-side algorithm updates from diverging too far from the global algorithm. This method makes the proposed system more robust to data heterogeneity than the FedAvg-based FL method. Experimental findings highlight that while centralized training may obtain the best accuracy, the FedAvg approach delivers the highest performance while ensuring data privacy. In contrast, the FedProx-based FL method gives more stable convergence in non-IID environments but may yield slightly lower accuracy than FedAvg. Overall, FedAvg strikes a strong balance between privacy, performance, and efficiency, whereas FedProx is preferable in environments with high data variability across clients.

Based on the above comparative analysis, it is evident that DL algorithms achieve superior performance under the FL approach. In a centralized approach, data is aggregated on a single server, which can lead to overfitting—particularly when the dataset is small or lacks diversity. Under such constraints, DL algorithms may struggle to capture rich, high-level features effectively. Conversely, FL addresses this issue by enabling local training on multiple decentralized clients, each possessing unique subsets of data. This decentralized strategy introduces a data heterogeneity approach. By aggregating model parameters from these diverse local algorithms, the global FL algorithm becomes more robust and better at generalizing across different data distributions. Thus, FL not only ensures data privacy but also reduces overfitting through its distributed and regularized learning process.

### 4.4 LIME result investigation

**Fig 7:**
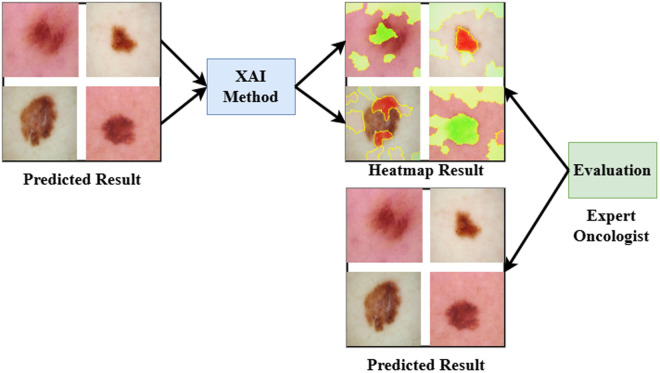
Block diagram of XAI-based LIME method to interpret the proposed framework.

**Fig 8:**
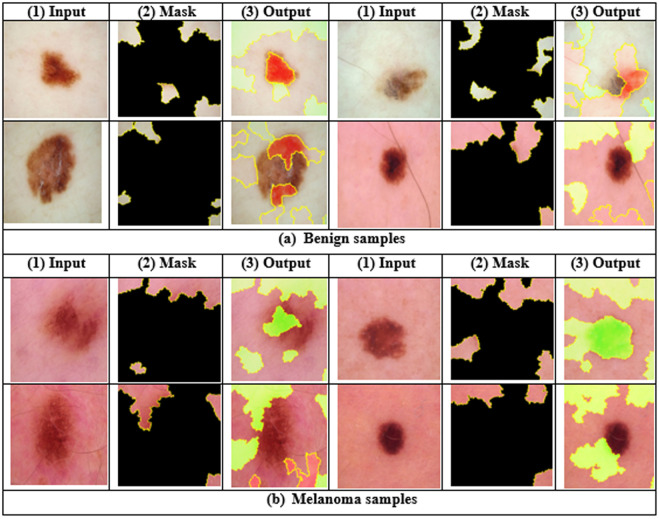
Result analysis of XAI-based LIME method: (a) produced mask and output for benign samples and (b) produced mask and output for melanoma samples.

The working principle of the XAI-based LIME method is illustrated in Fig 7 to help medical experts for final decision. In this experiment, we used LIME XAI method for model interpretability. In Fig 7, LIME makes segmented versions of samples by masking high-impact regions. Each segmented version is then provided into the DL algorithm to find prediction scores. Then LIME builds segmented method based on these prediction scores and highlights the high-impact pixels of the original data. Thus by highlighting these high-impact pixels, LIME method shows how a DL algorithm makes final prediction for a disease diagnosis. Fig 8 exhibits some examples of generated XAI result by LIME to interpret DL algorithm: (1) input data, (2) generated mask by LIME, and (3) segmented or output data.

**Fig 9:**
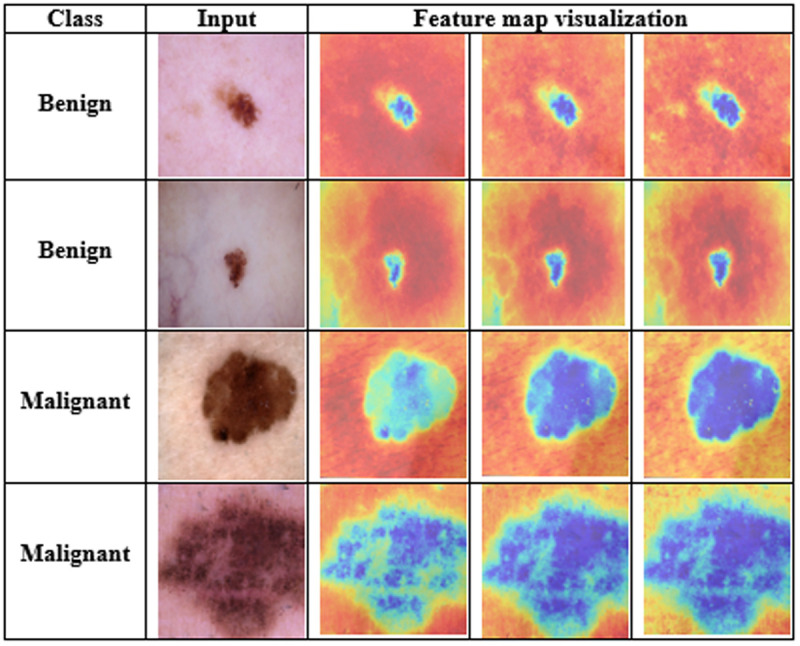
Feature map visualization of the last convolutional layer of the proposed model.

In Fig 8, LIME segments the predicted data into many small parts, where each part is connected to others with the same color. This algorithm highlights the high-resolution pixels to help oncologists make final decisions. Here, the red region highly represents the probabilities of a benign cancer area, while the green color indicates the melanoma cancer region. Fig 9 visualizes the feature map (FM) extracted from the last convolutional layer of the proposed model. In Fig 9, the blue regions indicate the low activation areas, whereas the yellow and red regions show high activation areas. The consistent activation patterns across multiple FMs indicate that the model is effectively capturing the distinguishing characteristics of the lesion.

In this manuscript, six advanced DL algorithms are trained on ISIB2016 and ISIB2017 datasets to test the classification performance of these algorithms. After that, these algorithms are trained under the FL platform for data privacy and improving the classification results. The FL approach not only ensures data privacy but also improves the classification reports. The classification reports of our proposed method are compared with other existing methods in Table 12. From Table 12, the proposed model outperforms existing approaches from previous studies. Compared to the results in [24], our method achieves an accuracy improvement of 11.61% on the ISBI2016 dataset and 13.53% on the ISBI2017 dataset. The study by [22] reported the second-highest accuracy of 88.02% using a hybrid CNN + SVM approach. In contrast, [24] recorded the lowest accuracy of 80.47%, highlighting the substantial performance gap addressed by our proposed method.

**Table 12:** Comparison of the suggested method with existing methods on the ISBI2016 and ISBI2017 datasets. Here, A indicates accuracy, P indicates precision, R indicates recall, S indicates specificity and F indicates F1-score.

				Accuracy difference	
Ref.	Dataset	Algorithm	Results (%)	ISBI2016	ISBI2017
[12]	ISBI2016	Deep residual network	R (54.7), P (62.4), A (85.5)	+6.58%	+8.5%
[25]	ISBI2016	CNN + FV	A (86.54)	+5.54%	+7.46%
[24]	ISBI2016	MLP+DenseNet121	A (80.47)	+11.61%	+13.53%
[29]	ISBI2016	DenseNet201 + MobileNet + SVM	A (88.02)	+4.06%	+5.98%
[14]	ISBI2016	VGG16	R (78.66), P (79.74), A (81.33)	+10.75%	+12.67%
[26]	HAM1000	DenseNet201	P (78.5), S (96), A (82.9)	+9.18%	+11.1%
Work1	ISBI2016	FL + VGG16	A (92.08), F (76.92), S (98.36), P (90.91)	Baseline	
Work2	ISBI2017	FL + VGG16	A (94), P (96.72), S (98.47), F (90.77).	Baseline	

Although the proposed approach demonstrates excellent performance, it still has several limitations: 1) The model’s performance could be further enhanced by incorporating real-world clinical datasets; 2) it lacks mechanisms to effectively address real-time data security and privacy concerns; and 3) the current study is only applicable for binary classification (benign or melanoma).

## 5. Conclusion and future scope

Skin cancer (SC) continues to pose a significant global health concern, making early and accurate diagnosis essential for effective treatment. This study explores the artificial intelligence-based deep learning (DL) algorithms to improve the classification of SC. To address key issues related to data security and limited data availability in the healthcare sector, we present a federated learning (FL) approach with a DL algorithm that enables collaborative model training without sharing sensitive information. Our simulated outcomes confirm the dependability of the proposed framework across diverse datasets. The proposed framework obtained 92.67% accuracy using the DenseNet169 algorithm on the ISBI2016 dataset and 83.3% accuracy using the same algorithm on the ISBI2017 dataset. Moreover, in the FL platform with multiple clients, the proposed framework provided accuracy levels as high as 92.08% using the VGG16 algorithm on ISBI2016 and 94% accuracy using the same algorithm on ISBI2017. This outstanding performance emphasizes the practicality of deploying AI in a data privacy and distribution manner.

In the future, we will expand the proposed framework to more diverse and large datasets, incorporating it into real-world clinical practices and enhancing the model’s interpretability. These advancements will be helpful for building trust in AI-assisted diagnostics and ensuring responsible and effective use in healthcare settings. This research will highlight the transformative potential of AI, particularly the FL approach, in reshaping the future of medical diagnostics and patient care.
